# P-1171. Assessment of the Fosfomycin 200 µg Disk Diffusion against Resistant Enterobacterales: Data from the Intravenous Fosfomycin (IV-FOS) Clinical Trial Program for Complicated Urinary Tract Infection

**DOI:** 10.1093/ofid/ofaf695.1364

**Published:** 2026-01-11

**Authors:** jason M Pogue, Keith S Kaye, Surya Chitra, rolf Wagenaar, Mauricio Rodriguez, Judith N Steenbergen

**Affiliations:** University of Michigan, College of Pharmacy, Ann Arbor, MI; Rutgers Robert Wood Johnson Medical School, New Brunswick, NJ; Savio Group Analytics, Hockessin, Delaware; SMAC, Gladstone, New Jersey; Meitheal Pharmaceuticals, Austin, TX; Scientific and Medical Affairs Consulting, LLC, Washington Crossing, Pennsylvania

## Abstract

**Background:**

Intravenous fosfomycin (IV-FOS) is an injectable epoxide antibiotic under evaluation by the U.S. Food and Drug Administration (FDA) for the treatment of cUTI/AP with activity against drug-resistant Enterobacterales. Appropriate use of antibiotics relies on accessible and accurate susceptibility testing. Agar dilution, the gold standard for fosfomycin susceptibility testing, is resource intensive. Comparatively, disk diffusion (DD) is a low cost and accessible method of susceptibility testing for clinical laboratories. This study evaluated commercially available 200 µg fosfomycin disks (supplemented with 50 µg glucose-6-phosphate (G6P)) against clinical isolates including drug-resistant subgroups.

**Figure 1.**
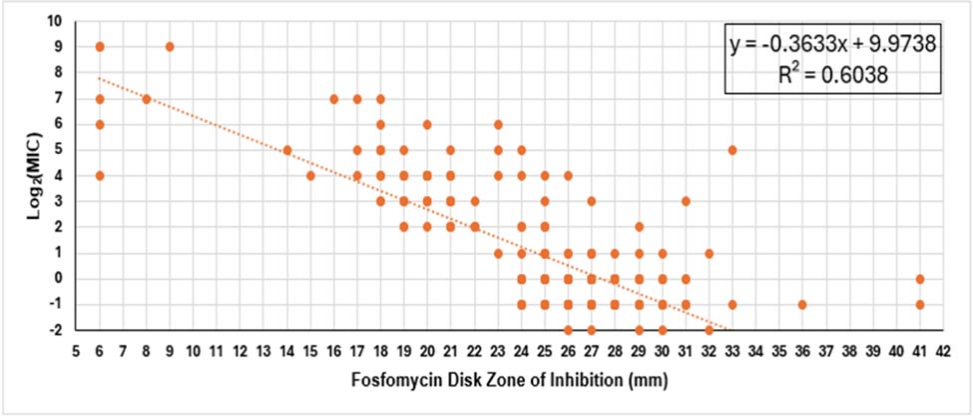
Regression analysis correlating zones of inhibition (mm) with MICs determined by CLSI agar dilution method (µg/mL).

**Methods:**

Fosfomycin was tested against a collection of Enterobacterales isolates from clinical and preclinical studies. 239 clinical isolates were tested (n = 175 clinical trial isolates, n = 64 pre-clinical isolates). DD was performed using a 200 μg fosfomycin disk supplemented with 50 μg of G6P. Minimum inhibitory concentrations (MIC) were determined by agar dilution in the presence of G6P according to Clinical and Laboratory Standards Institute (CLSI) methodologies. To determine the correlation between the DD and the agar dilution methods, zone diameters were plotted against their respective MICs and a linear regression analysis was performed.

**Results:**

Most isolates were *E. coli* (n = 146) [MIC range: 0.25 to 32 g/mL] followed by *K. pneumoniae* (n = 31) [MIC range: 4 to ≥ 512 g/mL]. A scattergram of MIC to zone diameter correlates for fosfomycin is shown in Figure 1. Good correlation between the two methods was observed (Pearson’s correlation, R = -0.78), which is similar to the correlation reported for recently approved antibiotics given DD breakpoints from the FDA.

**Conclusion:**

DD is a reliable and accessible method of susceptibility testing for fosfomycin. These studies support the use of commercially available fosfomycin disks to perform susceptibility tests with accurate and reproducible methods. Once MIC breakpoints are set by the FDA, these data can be utilized to define the corresponding DD breakpoints. The implementation of DD may reduce inappropriate therapy by enabling timely and reliable fosfomycin susceptibility results.

**Disclosures:**

jason M. Pogue, PharmD, Entasis: Advisor/Consultant|Entasis: Grant/Research Support|GlaxoSmithKline: Advisor/Consultant|Melinta: Grant/Research Support|Merck: Advisor/Consultant|Merck: Grant/Research Support|Shionogi: Advisor/Consultant|Shionogi: Grant/Research Support Keith S. Kaye, MD, MPH, AbbVie: Advisor/Consultant|GSK: Advisor/Consultant|Merck: Advisor/Consultant|Shionogi: Advisor/Consultant rolf Wagenaar, MS, Meitheal: Advisor/Consultant Mauricio Rodriguez, PharmD, MS-HEOR, BCCCP, BCIDP, Meitheal Pharmaceuticals: employee Judith N. Steenbergen, PhD, AcurX: Advisor/Consultant|Basilea: Advisor/Consultant|Bioversys: Advisor/Consultant|Clarametyx: Advisor/Consultant|Eagle Pharmaceuticals: Advisor/Consultant|F2G: Advisor/Consultant|Genentech: Advisor/Consultant|Innoviva: Advisor/Consultant|Meitheal: Advisor/Consultant|Melinta: Advisor/Consultant|Neuraptive: Advisor/Consultant|Neuraptive: Advisor/Consultant|Roche: Advisor/Consultant|Wockhardt: Advisor/Consultant

